# Analysing the Large Decline in Coronary Heart Disease Mortality in the Icelandic Population Aged 25-74 between the Years 1981 and 2006

**DOI:** 10.1371/journal.pone.0013957

**Published:** 2010-11-12

**Authors:** Thor Aspelund, Vilmundur Gudnason, Bergrun Tinna Magnusdottir, Karl Andersen, Gunnar Sigurdsson, Bolli Thorsson, Laufey Steingrimsdottir, Julia Critchley, Kathleen Bennett, Martin O'Flaherty, Simon Capewell

**Affiliations:** 1 Icelandic Heart Association, Kopavogur, Iceland; 2 University of Iceland, Reykjavik, Iceland; 3 Landspitali University Hospital, Reykavik, Iceland; 4 Institute of Health and Society, University of Newcastle, Newcastle upon Tyne, United Kingdom; 5 Department of Pharmacology and Therapeutics, Trinity Centre for Health Sciences, St James's Hospital, Dublin, Ireland; 6 Division of Public Health, University of Liverpool, Liverpool, United Kingdom; University of Michigan, Canada

## Abstract

**Background:**

Coronary heart disease (CHD) mortality rates have been decreasing in Iceland since the 1980s. We examined how much of the decrease between 1981 and 2006 could be attributed to medical and surgical treatments and how much to changes in cardiovascular risk factors.

**Methodology:**

The previously validated IMPACT CHD mortality model was applied to the Icelandic population. The data sources were official statistics, national quality registers, published trials and meta-analyses, clinical audits and a series of national population surveys.

**Principal Findings:**

Between 1981 and 2006, CHD mortality rates in Iceland decreased by 80% in men and women aged 25 to 74 years, which resulted in 295 fewer deaths in 2006 than if the 1981 rates had persisted. Incidence of myocardial infarction (MI) decreased by 66% and resulted in some 500 fewer incident MI cases per year, which is a major determinant of possible deaths from MI. Based on the IMPACT model approximately 73% (lower and upper bound estimates: 54%–93%) of the mortality decrease was attributable to risk factor reductions: cholesterol 32%; smoking 22%; systolic blood pressure 22%, and physical inactivity 5% with adverse trends for diabetes (−5%), and obesity (−4%). Approximately 25% (lower and upper bound estimates: 8%–40%) of the mortality decrease was attributable to treatments in individuals: secondary prevention 8%; heart failure treatments 6%; acute coronary syndrome treatments 5%; revascularisation 3%; hypertension treatments 2%, and statins 0.5%.

**Conclusions:**

Almost three quarters of the large CHD mortality decrease in Iceland between 1981 and 2006 was attributable to reductions in major cardiovascular risk factors in the population. These findings emphasize the value of a comprehensive prevention strategy that promotes tobacco control and a healthier diet to reduce incidence of MI and highlights the potential importance of effective, evidence based medical treatments.

## Introduction

Life expectancy in Iceland is increasing and the gap between men and women is narrowing. Compared with other nations, life expectancy for Icelandic men is the highest in Europe at 79.6 years, and for Icelandic women comes seventh at 83.0 years [1]. The successful lowering of premature deaths before the age of 75 can be largely attributed to the decline in coronary heart disease (CHD) death rates, which between 1981 and 2006 fell 80% in men and women. This means that 295 fewer cardiac deaths (ICD10: I20–I25) occurred than would have been expected for this population if 1981 mortality rates had persisted [1].

These 295 fewer deaths thus represented 69% of the 426 fewer deaths resulting from the large decrease in total mortality rates in Iceland between 1981 and 2006. In the age group 25–74 years, this involved a 47% fall in men and a 30% fall for women.

The Icelandic Heart Association (IHA) was established in 1966, and in 1967 it began the Reykjavik Study, a prospective population based cardiovascular survey [2] An earlier report from the Reykjavik Study described the increase in mortality in Icelandic men until the late 1970s and then the decrease during 1981 – 1986, along with corresponding changes in risk factor levels and food consumption.[3]

Since 1996, Capewell and colleagues have developed and refined a CHD mortality model called IMPACT. This model has been used to explore the recent declines in CHD mortality in diverse populations, and to comprehensively assess the potential contribution of medical treatments and risk factor changes. The model was validated against the actual mortality falls observed in England, Scotland, Ireland New Zealand, Finland, Sweden, and the USA. [4], [5], [6], [7], [8], [9], [10] It was also used to assess substantial increases recently seen in China.[11] The model was also able to calculate the life-years gained by different interventions [12], [13], and to perform cost-effectiveness analyses.[14]

The IMPACT model was subsequently used to address policy questions, estimating the additional deaths which could potentially be prevented, either by increasing the uptake of appropriate treatments in eligible patients,[15] or alternatively, by further modest reductions in specific risk factors.[16], [17] Results using the IMPACT model were generally consistent with other studies using diverse methodologies in different countries.[18], [19], [20], [21]

In this paper, we applied the IMPACT model to Icelandic data to explain the very large decline in coronary heart disease mortality in Iceland between 1981 and 2006 for men and women aged 25–74 years.

## Methods

### The IMPACT CHD mortality model

IMPACT was used to combine and analyse data on the Icelandic population (total 304,334 in 2006) aged 25–74 years (177,364 in 2006), stratified by age and sex. The Model includes comprehensive coverage of all standard evidence-based medical and surgical treatments used for coronary heart disease, quantifying the use and effectiveness of specific treatments. The model also estimates the mortality effects of changes in the major population risk factors for coronary heart disease: smoking, total cholesterol, systolic blood pressure, body mass index, diabetes and physical activity.

We therefore incorporated data for men and women aged 25 to 74 years in Iceland detailing:

a) CHD patient numbers (categorised by disease sub-group): Hospital discharge Records on myocardial infarction (MI), percutaneous coronary intervention (PCI) and coronary artery bypass grafting (CABG) for the population of Iceland available from 1981 to 2006 and maintained by the Icelandic Heart Association.

b) use of specific medical and surgical treatments from Landspitali University Hospital (LSH). It is the only hospital in Iceland providing PCI and CABC procedures and almost none are performed abroad. The hospital is also the primary treating facility for 80% of all myocardial infarctions.

c) effectiveness of specific cardiological treatments,

d) population trends in major cardiovascular risk factors (smoking, total cholesterol, systolic blood pressure, obesity, diabetes and physical activity), based on population studies by the Icelandic Heart Association.

*and*

e) effectiveness of specific risk factor reductions based on published meta-analyses.

Data from other sources were used only in rare instances. Data sources are summarised in Table 1, and are detailed in the Supplementary Appendix S1.

**Table 1:** Main Data Sources for the Parameters Used in the Iceland IMPACT Model.

Data	1981	2006
Population statistics		
Population, deaths, CHD mortality	Statistics Iceland	Statistics Iceland
**Surgical and medical treatments**		
Number of patients admitted yearly: MI, AP, HF, and treated CABG, PCI	Icelandic Heart Association, Landspitali - National Hospital	Icelandic Heart Association, Landspitali - National Hospital
Cardiopulmonary resuscitation in thecommunity	Assume zero	Landspitali - National Hospital
Hospital Resuscitation	Assume zero	Landspitali - National Hospital
Thrombolysis	Assume zero	Landspitali - National Hospital
Aspirin	Assume zero	Landspitali - National Hospital
Beta blockers	Assume zero	Landspitali - National Hospital
Warfarin	Assume zero	Landspitali - National Hospital
Heparin	Assume zero	Landspitali - National Hospital
ACE inhibitors	Assume zero	Landspitali - National Hospital
Statins	Assume zero	Landspitali - National HospitalREFINE Reykjavik Study(data from 2005–2007)
**Risk factors**		
Smoking	Reykjavik Study – Stage III, IV, MONICA Survey(data from 1979 – 1983)	REFINE Reykjavik Study(data from 2005–2007)
Hypertension prevalence		
Systolic blood pressure		
Cholesterol		
Physical activity		
Obesity (BMI)		
Diabetes		

### Deaths prevented or postponed in 2006

The number of CHD deaths expected in 2006 if the mortality rates in 1981 had persisted was calculated by indirect age standardization by sex and 10-year age groups from age 25–74, using 1981 as the base year. The CHD deaths actually observed in 2006 were then subtracted to give the difference in CHD deaths between 1981 and 2006 (Table 2).

**Table 2:** Population numbers and CHD deaths in Iceland 1981 and 2006 with estimated fall in CHD deaths in 2006 if 1981 rates persisted.

			CHD deaths		CHD deaths if 1981 rate persisted	
**Men**	**1981**	**2006**	**1981**	**2006**	**2006**	**Fall in deaths**
25–34	18638	23135	1	0	1	1
35–44	12482	22419	6	2	11	9
45–54	11012	21594	19	7	37	30
55–64	9149	15097	59	18	97	79
65–74	6154	8715	101	35	143	108
**TOTAL**	**57,435**	**90,960**	**186**	**62**	**290**	**228**
**Women**	**1981**	**2006**	**1981**	**2006**	**2006**	**Fall in deaths**
25–34	17449	21650	0	0	0	0
35–44	12035	21084	0	0	0	0
45–54	10945	19873	1	2	2	0
55–64	9325	14428	9	3	14	11
65–74	6962	9369	51	12	69	57
**TOTAL**	**58,697**	**88,410**	**61**	**17**	**84**	**67**
**Total**			**247**	**79**	**374**	**295**

### Mortality reductions attributable to treatments

The IMPACT Model aims to be comprehensive, and includes all standard medical and surgical treatments provided in 1981 and 2006. These interventions are listed in Table 3 and included all the treatments considered in earlier versions of the IMPACT Model, plus primary angioplasty and stenting for myocardial infarction, statins for primary prevention, platelet IIB/IIIA inhibitors and clopidogrel for unstable angina, and spironolactone and beta-blockers for heart.

**Table 3:** Estimated deaths prevented or postponed by medical or surgical treatments in Iceland 2006.

TREATMENTS	Patients Eligible	Best estimate (no. of deaths)	Min	Max	Best estimate (% of total)	Min (%)	Max (%)
Acute MI	**247**	9	1	10	3.2	0.2	3.3
Unstable Angina	**358**	7	3	9	2.3	1.2	2.9
2′ Prev Post AMI	**1649**	14	6	29	4.9	2.1	9.7
2′ Prev Post CABG/Ax	**1147**	10	4	19	3.3	1.3	6.6
Chronic Angina	**3990**	8	5	18	2.9	1.8	6.0
Hospital Heart Failure	**81**	5	1	3	1.5	0.3	1.0
Community H Failure	**956**	12	5	14	4.0	1.6	4.7
Hypertension Treatment	**37913**	7	1	14	2.2	0.2	4.6
TOTAL Statins, Gem & Niacin 1′ prevention	**92283**	1	1	3	0.5	0.2	1.0
Total Treatment		**73**	**23**	**117**	24.7	7.9	39.8

To avoid double counting, potential overlaps between different groups of patients were identified and adjustments were made using previously tested methods.[6]

The potential effect of multiple treatments in an individual patient was estimated using the Mant and Hicks cumulative relative benefit approach [22]:

Relative Benefit  = 1−[(1−Treatment A) × (1−Treatment B) × ••• × (1−Treatment N).

A number of effective therapies were already in limited use in 1981. These included CABG surgery, cardiopulmonary resuscitation, beta-blockers for acute myocardial infarction, and therapy for moderate and severe hypertension (defined as a diastolic blood pressure >105 mmHg). Precise patient data for some of these interventions, such as CABG, and eligible hypertensives, were obtained from the data sources detailed above. Others were estimated after consultation with cardiologists in practice in 1981.

### Mortality reductions attributable to changes in risk factors

Two approaches were used to calculate the numbers of deaths prevented or postponed as a result of changes in risk factors.

a) We used a **regression approach** for systolic blood pressure, cholesterol, and body mass index (BMI). The number of deaths prevented or postponed as a result of the change in the value for each of these risk factors (Table 4) was estimated as the product of three variables: the number of CHD deaths observed in 2006 if rates from 1981 persisted (the base year), the subsequent reduction in that risk factor and the regression coefficient quantifying the change in mortality from coronary heart disease per unit of absolute change in the risk factor (Supplementary Appendix S1). For example, in 2006 there were 12 CHD deaths among 9369 women aged 65–74; the expected number of deaths would have been 69 if rates from 1981 persisted. Between 1981 and 2006 the mean systolic blood pressure in this group decreased by 7.9 mmHg. The largest meta-analysis showed an estimated age- and sex-specific reduction in mortality of 50% for every 20 mmHg reduction in systolic blood pressure, generating a logarithmic coefficient of –0.032.[23] The number of deaths prevented or postponed as a result of the change observed in Iceland was then estimated as:[6]

**Table 4:** Deaths from coronary heart disease prevented or postponed as a result of changes in population risk factors in Iceland 1981 – 2006.

	Risk factor levels		Risk factor change		RR or coef	DPP Best	Min;Max	Fall (%)	Min;Max(%)
	1981	2006	Absolute	Relative(%)					
Smoking (PARF)	46.5%	22.6%	23.8	−51.3	2.52	65	52;80	22.0	17.6;26.4
Systolic BP(regression)	126.3	121.2	5.1	−4.0	−0.03	65	46;90	22.0	15.5;31.0
Cholesterol (regression)	6.01	5.14	0.87	−14.5	−0.68	95	64;115	31.6	21.7;39.5
Physical Inactivity (PARF)	76.8%	53.8%	23.0	−30.0	1.27	15	13;20	5.4	4.3;6.5
BMI (regression)	25.0	27.0	−2.0	7.8	0.03	−15	−7; −20	−4.4	−2.5; −6.9
Diabetes (PARF)	1.7%	3.6%	−1.9	107.4	1.93	−15	−9; −20	−4.6	−3.2, −6.3
**Total Risk** **Factors**						**210**	158;265	**72.6**	53.5;90.2

Number of deaths prevented  = (1−e^(coefficient x change)^ ) x expected deaths in 2006

 = (1−e^ (−0.032×7.9)^ ) ×69 = 15.

b) A **population-attributable risk fraction approach** was used to determine the impact of changing prevalence of smoking, diabetes and physical inactivity. The population-attributable risk fraction was calculated conventionally as (P x (RR-1))/(1+P x (RR-1)) where P is the prevalence of the risk and RR is the relative risk for CHD mortality associated with that risk factor. The number of deaths prevented or postponed was then estimated as the number of expected deaths from coronary heart disease in 2006 if 1981 rates persisted (the base year) multiplied by the difference between the population-attributable risk fraction in 1981 and that in 2006. For example, the prevalence of diabetes in men aged 65–74 years increased from 8.7% in 1981 to 14.6% in 2006. The expected number of deaths would have been 143 in 2006 if rates from 1981 persisted. Given a relative risk of 1.93[9], the population-attributable risk fraction increased from 0.075 to 0.119. Additional deaths in 2006 attributable to an increased prevalence of diabetes were therefore calculated as follows:[6]

Additional deaths in 2006 = (143) × (0.119 – 0.075)  = 6.4.

Because all the regression coefficients and relative risks for each risk factor were independent, being taken from multivariate analyses, we assumed that there was no further synergy between the major risk factors or between treatment and risk factor sections of the model.

The numbers of deaths prevented or postponed as a result of risk factor changes were systematically quantified for each specific age group in men and women to account for potential differences in effect. It was assumed that lag times between the change in the risk factor rate and event rate change would be relatively unimportant over a period of 25 years. [6], [24], [25]

### Sensitivity Analyses

Because of the uncertainties surrounding many of the values, multi-way sensitivity analyses were performed using Brigg's analysis of extremes method.[26]. Minimum and maximum mortality reductions were generated for therapeutic effectiveness, using 95% confidence intervals for relative risk values obtained from the most recent meta-analyses. The minimum and maximum plausible values for the remaining key parameters reflected the quality of the available data. Current default values in the IMPACT Model are: eligible patient numbers +10%, treatment uptake +20%, and compliance +25%. Corresponding sensitivity analyses were constructed for risk factors, the key parameters being change in risk factor levels, the CHD death numbers in 1981, the base year and the β coefficient or relative risk.

## Results

### Mortality trends 1981–2006

Between 1981 and 2006 in the age range 25–74, the standardized mortality rate for coronary heart disease (ICD-9 410-414) decreased 79% from 324 to 68 for every 100,000 men and decreased 82% in women, from 431 to 88 per 100,000 (direct method , Icelandic census 1981) (3,4). This fall resulted in 295 fewer deaths observed in 2006 (Table 2).

**Figure 1:**
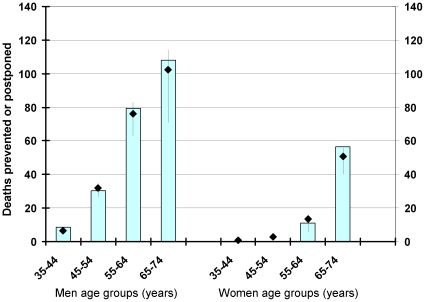
Model fit comparing observed and estimated mortality falls in each age group. *(diamonds  =  best estimate, bars maximum and minimum estimate).*

The mortality fall estimated by the IMPACT model showed a reasonably good fit in specific age and sex groups (Figure 1).

### Mortality reduction attributable to medical and surgical treatments

The model suggested that treatments together accounted for approximately 73 fewer deaths in 2006 *(minimum estimate 23, maximum 117).* Thus, approximately one quarter (25%) of the 295 fewer deaths was attributable to treatments in individuals: including some 8% to secondary prevention, 6% to heart failure treatments, 6% to initial treatments of acute coronary syndrome, 2% to hypertension treatments, 0.5% to statins for primary prevention, and revascularisation accounted for approximately 3% (principally angioplasty in patients with AMI or unstable angina.) (Table 3).

### Mortality reduction attributable to risk factor changes

Risk factor changes together accounted for approximately 215 fewer deaths in 2006 *(minimum estimate 159, maximum estimate 275).* (Table 4).

The largest contribution came from reductions in cholesterol (95 fewer deaths), systolic blood pressure (65) and smoking (also 65).

Almost three quarters of the mortality decrease (73%) was thus attributable to population risk factor reductions (principally cholesterol, 32%; smoking, 22% and systolic blood pressure, 22%, with 5% from increase in physical activity).

Adverse trends were seen for diabetes prevalence (increasing from 1.7% to 3.6%) and obesity (mean BMI rising from 25.0 to 27.0). These rises together generated approximately 30 additional deaths *(minimum estimate 16, maximum estimate 40).*

### Sensitivity analyses

**Figure 2:**
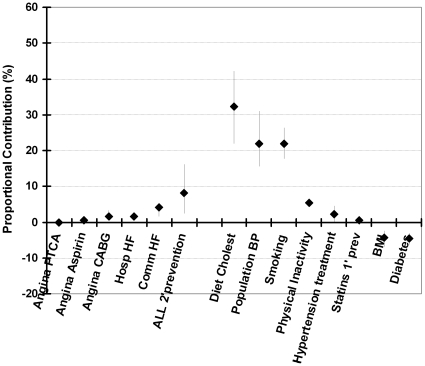
The proportional contributions of specific risk factor treatments and risk factor changes to the overall decrease in CHD mortality in Iceland between 1981 and 2002. The bars show the observed deaths in each age group, with diamonds being the best model estimate, and vertical lines the extreme minimum and maximum estimates in the sensitivity analysis.

Treatments together accounted for approximately 25% of the mortality fall *(minimum estimate 8%, maximum 40%).* Risk factors contributed the remaining 73% *(minimum 54%, maximum 93%)* with approximately 2.7% of the fall not being explained by the model. The mortality reductions attributable to specific treatments were thus consistently smaller than those achieved by reductions in the major risk factors (Figure 2).

## Discussion

Between 1981 and 2006, coronary heart disease mortality rates in Iceland decreased by 80% in men and women aged 25 to 74 years. This is one of the largest falls recorded in Western populations, averaging 3.2% per year over two and a half decades. It compares well with Finland (4.2% per annum), Sweden (3.3%) [10], the UK (2.8%) [27], and the USA (2.5%) [9].

This huge mortality fall resulted in 295 fewer deaths in 2006 in Iceland. Approximately one quarter of the mortality decrease (25%) was attributable to treatments in individuals. Useful contributions came from secondary prevention, heart failure treatments, and initial treatments of acute coronary syndrome much as in Sweden (10) and Finland (7). The contribution from revascularizations was modest, reflecting a relatively small number of eligible patients and limited mortality benefits in stable angina[25]. However, the improvement in quality of life is important and well recognized [28].

Risk factor changes together accounted for approximately 210 fewer deaths in 2006 *(minimum estimate 158, maximum estimate 265).* Thus over 70% of the large mortality decrease was attributable to population risk factor reductions. The main contributions came from secular falls in cholesterol, (32%); systolic blood pressure, (22%) and smoking, (22%). A further 5% came from increases in physical activity, in line with trends in Scandinavia (7, 10).

**Figure 3:**
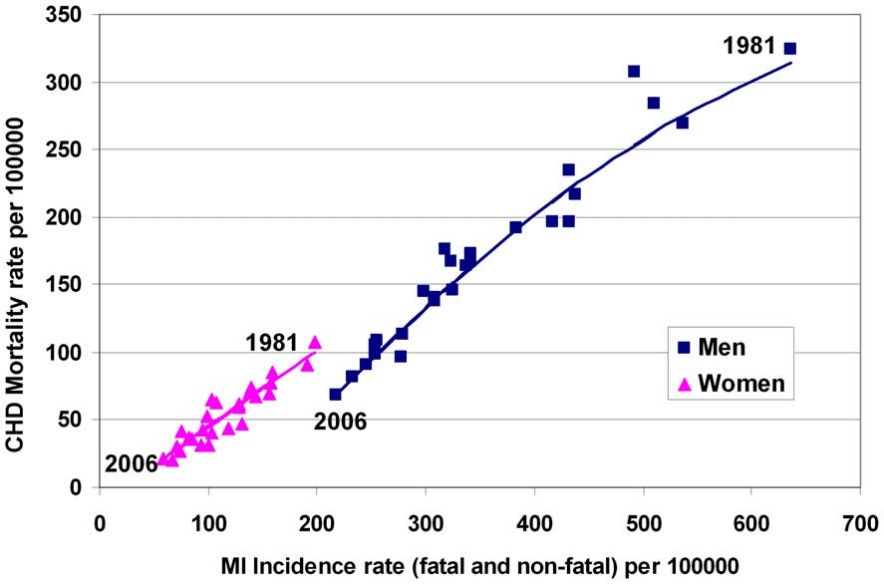
CHD mortality rates and MI incidence rates in Iceland between 1981 and 2006 for men and women of age 25–74. The rates have been declining along the superimposed trend lines.

This mortality fall in turn reflected a large fall of some 500 fewer incident MI cases in 2006 (than if 1981 rates persisted): The total MI incidence rate declined by 66% for men and women between 1981 and 2006 (Figure 3). Any further reduction in CHD deaths is mainly going to be obtained through further reduction in the incidence of MI. Therefore, in spite of the dramatic falls in major cardiovascular risk factors, there remains an additional potential for further improvement. Having used the IMPACT Model to successfully quantify the Icelandic population in 2006, we should now explore the further reduction in mortality that might be achieved. For instance if mean total cholesterol levels were decreased to 5.0 or even 4.5 mmol/l, systolic blood pressure levels to 115 mmHg and smoking prevalence to just 10% of adults.[29]

As in Finland, Sweden China and elsewhere, cholesterol was the most powerful risk factor; this remains true whether considered as sub-fractions or in total. In middle aged adults, a 1 mmol/l reduction will reduce CHD mortality rates by approximately one third[30]. Cholesterol levels are powerfully influenced by diet, particularly the relative intake of (“good”) polyunsaturated fats from vegetable oils, nuts and fish versus (“bad”) saturated fats from meat and dairy products) and (“very bad”) transfats from hydrogenated fatty acids concealed in junk food, processed food, cakes and cookies.[31]

The large (0.87 mmol/l) fall in cholesterol between 1981 and 2006 reflects major changes in the Icelandic diet following the issue of Dietary Goals for Icelanders, published by the National Nutrition Council in the fall of 1986 and subsequent approval of a National Food and Nutrition Policy by both the executive and parliamentary branch of the Icelandic government in May 1989. The National policy is based on the dietary goals where the reduction of saturated fat, mainly from milk and dairy products, butter, lamb and margarine, is greatly emphasized. The food policy and the dietary goals have greatly influenced nutrition education and awareness in the country.

Icelandic food supply data clearly demonstrate the subsequent changes. In the 1970s, the diet was characterized by high consumption of whole milk and dairy products, margarine, butter, lamb, mutton and fish. However, between 1980 and 2006 there was a 73% drop in whole milk and dairy consumption (from 238 kg/person/year to 64 kg/person/year) and the supply of lamb and mutton decreased by 50% (from 47 kg/person/year to 24 kg/person/year). The lamb supply has been replaced by other meat products, mainly poultry and pork. Further, the supply of margarines made from hydrogenated fats and used for cooking and baking, has plummeted by 73% (from 11.7 kg/person/year to 3.2 kg/person/year), the largest drop occurring in the 1990s, when the most significant drop in cholesterol was also seen. From the 1990s, the consumption of total fat in percent energy (E%) calculated from the food supply statistics has decreased from 40E% to 36E% . But more importantly, the composition of the fat has also changed from more saturated and *trans*-fatty acids to *cis*-unsaturated.[32] In contrast, statins played a relatively small role in lipid lowering at the population level for middle aged Icelanders up until 2006.[33]

Solid data on salt or sodium intake on a population level are not available. However during this period a gradual shift occurred from the traditional Icelandic foods dominated by salted and cured meats and fish, to fresh and frozen foods. The contribution of these changes to the fall in blood pressure can only be hypothesized.

Smoking prevalence fell substantially from 47% to 23% overall of which prevalence for non-cigarette smokers fell from 14% to under 3% in 2006. This reflects series of successful tobacco control initiatives including a ban on advertising tobacco in 1971, ban on smoking at workplaces in 1985, ban on selling tobacco to minors in 1996, culminating in legislation to ban smoking in public spaces in June 2007.

However, adverse trends were seen for obesity and diabetes, increasing mortality by 4% and 5% respectively. This mirrors similar disappointing increases in the UK and USA. It mainly reflects excessive intake of calories not compensated by the amount of physical activity in daily life, (even though leisure physical activity levels increased).[34] Future policies will therefore need to focus on calorie restriction at individual, local and national levels.

### Strengths and limitations of this study

The best modelling studies have a number of potential strengths. They can transparently consider and simultaneously integrate large amounts of data from many sources. Explicit assumptions can then be tested by sensitivity analyses [6]. Yet modelling studies also have limitations. They represent crude simplifications of a complex reality. In particular, they depend on the variable extent and quality of data available on CHD risk factor trends and treatment uptakes. However, population data and hospital discharge registries in Iceland are particularly good and almost 100% complete. Since Iceland has almost no private hospitals and no private Cardiac Care Units (CCUs), the data cover more than 99% of our hospital patients, and clearly capture the majority of the Icelandic population. This, together with a long tradition of high quality national population surveys and registries, reduces the potential problem of needing to make assumptions about dubious data.

The number of people aged 25–74 in Iceland is small (about 180000 in 2006) and CHD deaths few (79 in 2006), which is reflected in large margins of errors. However, the drop in CHD deaths and MI has been uniform since 1981 in this agegroup and the estimates from the model are consistent with observed data (Figure 1).

The model included only those adults aged 25 to 74 years old because of very limited data in older groups. Elderly patients and women have been consistently underrepresented in a majority of clinical trials and surveys in cardiovascular disease[35]. This highlights the need for further work on cardiovascular risk factors in the elderly.

In conclusion, almost three quarters of the large coronary heart disease mortality decrease in Iceland between 1981 and 2006 was attributable to reductions in major cardiovascular risk factors in the population, (mainly decreases in total serum cholesterol, smoking and blood pressure levels). These findings emphasize the value of a comprehensive strategy that promotes a healthier diet and further tightens tobacco control. It also highlights the potential importance of effective evidence based medical treatments for all eligible patients.

## Supporting Information

Appendix S1 IMPACT, a validated, comprehensive coronary heart disease model. Supplementary appendix for the Icelandic model.
(0.51 MB DOC)
