# Supplementary material for: Analysing the Large Decline in Coronary Heart Disease Mortality in the Icelandic Population Aged 25-74 between the Years 1981 and 2006
Source: PLoS One. 2010 Nov 12;5(11):e13957. doi: 10.1371/journal.pone.0013957 (PMC2980472; doi:10.1371/journal.pone.0013957)
Supplement: Appendix S1 — IMPACT, a validated, comprehensive coronary heart disease model. Supplementary appendix for the Icelandic model. (0.51 MB DOC) [file pone.0013957.s001.doc]

| 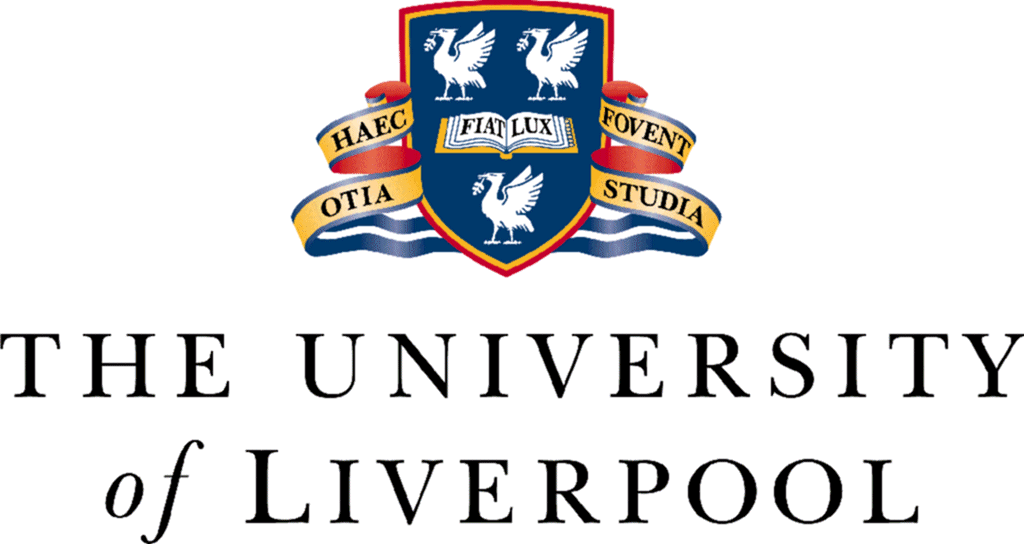  **IMPACT, A VALIDATED, comprehensive CORONARY HEART DISEASE MODEL**  **SUPPLEMENTARY APPENDIX**  **for the**  **Icelandic MODEL**  **Thor Aspelund, Vilmundur Gudnason, Bergrun T Magnusdottir, Karl Andersen, Bolli Thorsson, Gunnar Sigurdsson, Julia Critchley, Martin O’Flaherty & Simon Capewell,**  **February 2010** |
| --- |

**SUPPLEMENTARY APPENDIX FOR THE IMPACT MODEL**

|  |  | |
| --- | --- | --- |
| **Contents** |  | **Page** |
| Table 1. | The Icelandic IMPACT Model: Introduction detailed methodology and examples of deaths prevented or postponed (DPP) calculations | 3 |
| Table 2 | CHD mortality rates per 100,000 1981 and 2006 and difference in number of CHD deaths between 1981 and 2006 in men and women in Iceland | 3 |
| Table 3. | Main data sources for the parameters used in the Icelandic IMPACT Model | 13 |
| Table 4. | **Clinical efficacy of interventions:** relative risk reductions obtained from meta-analyses, and randomised controlled trials | 16 |
| Table 5. | **Data sources for treatment uptake levels in Iceland 2006:** Medical and surgical treatments included in the Model | 22 |
| Table 6. | Age-specific case fatality rates for each patient group | 24 |
| Table 7. | Specific beta coefficients for major risk factors: **Data sources, values and comments.** | 25 |
| Table 8. | **Relative risk values for CHD mortality: smoking, diabetes and physical inactivity** (Best, minimum and maximum estimates from InterHeart) | 27 |
| Table 9. | Icelandic IMPACT Model Risk Factor Methodology: Rationale for choice of regression or PARF approaches for specific risk factors | 28 |
| Table 10. | Assumptions and overlap adjustments used in the Icelandic IMPACT Model | 32 |

Table 1. The ICELANDIC IMPACT MODEL: INTRODUCTION and DETAILED METHODOLOGY

The tables included in this supplementary appendix document provide details about the methods that were used in creating the Icelandic IMPACT model. This model examines the effects of changes in treatments and risk factors trends on changes in mortality from coronary heart disease (CHD) among Icelandic adults aged 25-74 years (Table 2). Earlier versions of the IMPACT mortality model have been previously applied to data from Europe, New Zealand, USA and China.^1-8^ This cell-based mortality model, developed in Microsoft Excel, has been described in detail online and elsewhere.^1, 2, 9^

**Table 2.** CHD mortality rates per 100,000 1981 and 2006, and decrease in number of CHD deaths (n) in 2006 compared with 1981 baseline: men and women in Iceland

|  |  | **1981** | **2006** | |  | | |  |
| --- | --- | --- | --- | --- | --- | --- | --- | --- |
|  |  | **Rates per 100000** |  | **Rates per**  **100000** | |  | **Deaths prevented or postponed in 2006^a^** | |
| Men | | 323.8 |  | 68.2 | |  | 228 | |
|  | Women | 107.6 |  | 19.7 | |  | 67 | |
|  | Total |  |  |  | |  | 295 | |

^a^ The difference between observed and expected number of CHD deaths if 1981 rates had persisted.

**Changes in mortality rates from CHD, in Iceland 1981-2006**

Data sources used in examining the changes in mortality rates from 1981 to 2006 among Icelandic adults aged 25-74 years are shown in Table 3. Mortality rates from CHD were calculated using the underlying cause of death: International Classification of Diseases (ICD)-9 codes 410-414 and ICD-10 codes I20-I25. Both unadjusted and age-adjusted mortality rates were calculated. Age-standardization was done using the direct method based on the Icelandic projected 2006 population.

**Expected and observed number of deaths from CHD**

The data sources needed to estimate the expected and observed numbers of deaths from CHD for 2006 are shown in Table 3. The expected number of deaths from CHD in 2006 was calculated by multiplying the age-specific mortality rates from CHD in 1981 by the population counts for 2006 in that age-stratum. Summing over all age strata then yielded the expected numbers of deaths from CHD. The difference between the number of expected and observed number of deaths from CHD represents the mortality fall, the total number of deaths prevented or postponed (**DPPs**) from the combined changes in treatment patterns and risk factor prevalence.

**Treatments**

The treatment arm of the Model includes the following populations:

- Those hospitalized with an acute myocardial infarction (AMI)
- Patients admitted to the hospital with unstable angina pectoris (UAP)
- Community-dwelling patients who have survived an AMI
- Patients who have undergone revascularization procedure (Coronary Artery Bypass Grafting (CABG), or a Percutaneous Coronary Intervention (PCI)), with or without stent
- Community-dwelling patients with angina pectoris (no revascularization)
- Patients admitted to hospital with heart failure
- Community-dwelling patients with heart failure (no hospital admission).
- Hypertensive individuals eligible for hypotensive therapy
- Hypercholesterolaemic subjects eligible for cholesterol lowering therapy

The main data sources used to estimate the numbers of these groups are shown in Table 3. For each of the groups, we estimated the number of DPPs that were attributable to various treatments. A listing of the treatments that were considered in the model and the data sources used to estimate the percentages of patients receiving treatments are shown in Tables 4 and 5.

The general approach to calculating the number of DPPs from an intervention among a particular patient group was first to stratify by age and sex, then to multiply the estimated number of patients in the year 2006 by the proportion of these patients receiving a particular treatment, by the 1-year case-fatality rate, and by the relative reduction in the case-fatality rate due to the administered treatment. Sources for estimates of efficacy (relative risk reductions) are shown in Table 4. Sources for treatment uptakes are shown in Table 5. Age-specific case-fatality rates for each patient group are presented in Table 6.

We assumed that compliance (concordance)**,** the proportion of treated patients actually taking therapeutically effective levels of medication, was 100% among hospital patients, 70% among symptomatic community patients and 50% among asymptomatic community patients.^1, 4, 10, 11^ All of these assumptions were tested in subsequent sensitivity analyses.

**Example 1: estimation of DPPs from a specific treatment**

For example, in Iceland in 2006, about 76 men aged 55-64 were hospitalized with AMI in 2006 of whom approximately 87.6% were given aspirin. Aspirin reduces case-fatality rate by approximately 15%.^12^ The underlying 1-year case-fatality rate in these men was approximately 5.4%. the DPPs for at least a year were therefore calculated as

***Patient numbers x treatment uptake x relative mortality reduction x one-year case fatality***

***=*** [(76 x 0.876) x 0.054] x 0.15 = 0.5 deaths prevented or postponed

This calculation was then repeated

a) for men and women in each age group, and

b) incorporating a Mant and Hicks adjustment for multiple medications

c) using maximum and minimum values for each parameter in each group, to generate a sensitivity analysis (see below).

**Risk factors**

The second part of the IMPACT model involves estimating the number of coronary heart disease DPPs related to changes in cardiovascular risk factor levels in the population. The Icelandic IMPACT model includes total cholesterol, smoking, systolic blood pressure, body mass index (BMI), diabetes, and physical inactivity. Data sources used to calculate the trends in the prevalence (or mean values) of the specific risk factors are shown in Table 3.

Two approaches to calculating DPPs from changes in risk factors were used.

In the **regression approach**—used for systolic blood pressure, total cholesterol, and body mass index—the number of deaths from CHD occurring in 1981 (the base year) were multiplied by the absolute change in risk factor prevalence, and by a regression coefficient quantifying the change in CHD mortality that would result from the change in risk factor level. Natural logarithms were used, as is conventional, in order to best describe the log-linear relationship between changes in risk factor levels and mortality.

**Example 2: estimation of DPPs from risk factor change using regression method:**

***Mortality fall due to reduction in systolic blood pressure in women aged 55-64***

For example, among 14428 women aged 55-64 years, there were 14 CHD deaths in 1981, (the base year). Mean systolic blood pressure in this group then decreased by 8.72 mmHg (from 134.350 in 1981 to 125.630 mmHg in 2006). The largest meta-analysis reports an estimated age- and sex-specific reduction in mortality of 49 percent for every 20 mmHg reduction in systolic blood pressure, generating a logarithmic coefficient of –0.035.^13^

The number of deaths prevented or postponed in 2006 as a result of this change was therefore estimated as:

= (1-(EXP(coefficient*change))*deaths in 1981

= (1-EXP(-0.035*8.72))* 14 = 3.7

This calculation was then repeated

a) for men and women in each age group, and

b) using maximum and minimum values in each group, to generate a sensitivity analysis.

Data sources for the number of CHD deaths are shown in Table 3, sources for the population means of risk factors are shown in Table 3, and sources for the coefficients used in these analyses are listed in Table 7.

**Example 3: estimation of DPPs from risk factor change using PARF method.**

**Smoking in men aged 65-74 years**

The **population-attributable risk factor (PARF) approach** was used for smoking, diabetes, and physical activity. PARF was calculated conventionally as

**(P x (RR-1)) / (1+P x (RR-1))**

where P is the prevalence of the risk factor and RR is the relative risk for CHD mortality associated with that risk factor. DPPs were then estimated as the CHD deaths in 1981 (the base year) multiplied by the difference in the PARF for 1981 and 2006.

For example, the prevalence of smoking among men aged 65-74 years was 37.4% in 1981 and 12.9% in 2006. Assuming a Relative Risk of 2.52,^14^ the PARF was 0.362 in 1981 and 0.164 in 2006. The number of deaths prevented or postponed attributable to the decrease in smoking prevalence from 1981 to 2006 was therefore the CHD deaths in 1981, (143) * (0.362 - 0.164) = 28.3 DPPs

This calculation was then repeated

a) for men and women in each age group,

b) using maximum and minimum values in each group, to generate a sensitivity analysis

Data sources for the prevalence of risk factors and for the number of CHD deaths are shown in Table 3. Sources for the relative risks used in these PARF analyses are listed in Table 8. All come from the InterHeart study,^14^ the largest international study to provide independent RR values, adjusted for other major risk factors. The rationale for choosing the regression or PARF approaches for specific risk factors in the Icelandic IMPACT Model is detailed in Table 9.

**Other Methodological Considerations**

Several methodological issues will be discussed below. These include adjusting the relative reduction in case-fatality rate for patients receiving multiple treatments, establishing rules for avoiding double-counting individual patients who may fall into more than a single disease category (patient group), treatment overlaps, and sensitivity analyses.

**Polypharmacy Issues**

Individual CHD patients may take a number of different medications. However, data from randomized clinical trials on efficacy of treatment combinations are sparse. Mant and Hicks suggested a method to estimate case-fatality reduction by polypharmacy.^15^ This approach was subsequently endorsed by Yusuf^16^ and by Wald and Law.^17^

**Example 4: estimation of reduced benefit if patient taking multiple medications (Mant and Hicks approach)**

If we take the example **of secondary prevention following acute myocardial infarction,** good evidence (Table 4) suggests that, for each intervention, the relative reduction in case fatality is approximately: aspirin 15%, beta-blockers 23%, ACE inhibitors 20%, statins 22% and rehabilitation 26%. In individual patients receiving all these interventions, case-fatality reduction is very unlikely to be simply additive, i.e. not **106%** (15% + 23%+ 20% + 22% + 26%). This would clearly be impossible. The Mant and Hicks approach instead, suggests that having considered the 15% case fatality reduction achieved by aspirin, the next medication, in this case a beta-blocker, can only reduce the **residual** case fatality (100%-15%). Likewise, the subsequent addition of an ACE inhibitor can then only decrease the **remaining** case fatality, as a proportion this which will be 1 - [(1- 0.15) X (1-0.23)].

The Mant and Hicks approach therefore suggests that a **cumulative relative benefit** can be estimated as follows:

Relative Benefit = 1 - [(1-relative reduction in case-fatality rate for treatment A) X (1- relative reduction in case-fatality rate for treatment B) X ...X (1- relative reduction in case-fatality rate for treatment N). This approach has subsequently been endorsed by YUSUF (Lancet 2002) and by Wald and Law (BMJ 2005).

In considering appropriate treatments for AMI survivors, applying relative risk reductions (RRR) for aspirin, beta-blockers ACE inhibitors statins and rehabilitation then gives:

*Relative Benefit = 1 - [(1 –aspirin RRR) X (1 - beta-blockers RRR) X (1 - ACE inhibitors RRR) X (1- statins RRR) X (1- rehabilitation RRR)]*

= 1 - [(1- 0.15) X (1-0.23) X (1-0.20) X (1- 0.22) X (1- 0.26)]

= 1 - [(0.85) X (0.77) X (0.80) X (0.78) X (0.74)]

= **0.70 i.e. a 70% lower case fatality**

This represents a **34%** relative reduction (0.70/1.06) compared with the simple additive value of **106%.**

**Potential overlaps between patient groups: avoiding double counting**

The potential overlaps between CHD patient groups are shown in Table 10*.*

**Sensitivity Analyses**

Because of the uncertainties surrounding many of the values, multi-way sensitivity analyses were performed using Brigg’s analysis of extremes method^18^.

Minimum and maximum mortality reductions were generated for therapeutic effectiveness, using 95% confidence intervals for relative risk values obtained from the most recent meta-analyses or large randomised controlled trials. The minimum and maximum plausible values for the remaining key parameters, Patient numbers, treatment uptake and adherence, reflected the quality of the available data. Current default values in the IMPACT Model are: eligible patient numbers + 10%, treatment uptake + 20%, and compliance +25%. [13,25] Corresponding sensitivity analyses were constructed for risk factors, the key parameters being the β coefficient, relative risk, change in risk factor and CHD death numbers in 1981, the base year. An analysis of extremes was therefore performed whereby the maximum and minimum feasible values were fed in to the model. By multiplying through, the resulting product then generated maximum and minimum estimates for deaths prevented or postponed (Table below).

**Example: sensitivity analysis for AMI patients given aspirin**

An example of calculating lower and upper bound estimates for DPPs for treatment with aspirin among men aged 55-64 years who were hospitalized with an AMI is presented here. 95% confidence intervals from the meta-analysis were used for relative mortality reduction; lower and upper bound estimates for the other parameters were calculated as minus or plus 20% [except for treatment uptake that was capped at 99%]. Multiplying all the lower-bound estimates yielded the minimum [lower bound] estimate and multiplying the upper-bound estimates yielded the maximum [upper bound] estimate.

|  | **Patient**  **numbers** | **Treatment**  **Uptake** | **Relative**  **Mortality**  **Reduction^a^** | One year case fatality | **Deaths prevented**  **or postponed** |
| --- | --- | --- | --- | --- | --- |
|  | **A** | **B** | **C** | **D** | **(A x B x C x D)** |
| Best Estimate | 76 | 87.6% | 15% | 5.4% | 0.5 |
| Minimum estimate | 61 | 70.1% | 11%^a^ | 4.3% | 0.2 |
| Maximum estimate | 91 | 0.99 | 19%^a^ | 6.5% | 1.1 |

^a^lower and Upper 95% CI from the Antithrombotic Trialists’ Collaboration meta-analysis, ^12^ see Table 4.

This approach may be described as a “robust” approach for two reasons.

a) maximum and minimum values for each variable were deliberately forced to provide a wider range rather than a narrower one, e.g. relative mortality reduction +20% rather than say, +10%.

b) the resulting product, for instance the minimum estimate, was generated by assuming that the lowest feasible values all occurred at the same time, a most unlikely situation.

| Table 3. Main Data Sources for the Parameters Used in the Icelandic IMPACT Model | | |
| --- | --- | --- |
|  | **1981** | **2006** |
| Population statistics (number) | Statistics Iceland | Statistics Iceland |
| Deaths by age and sex (number) | Statistics Iceland | Statistics Iceland |
| CHD Mortality (rates) | Statistics Iceland  (ICD-9 codes 410-414) | Statistics Iceland  (ICD-10 codes I20-I25) |
| Number of patients admitted yearly | |  |
| Myocardial infarction:  ICD9: 410-414, ICD121-I25 | IHA | IHA |
| Angina pectoris:  ICD9: 413, ICD10: I20 |  | IHA |
| Heart failure: ICD9: 425.4, 425.5,  425.9, 428.0, 428.1 and 428.9 and  ICD10:I50. |  | IHA |
| **Number of patients treated with** |  |  |
| CABG: ICD-9 36.1  3066,3067, 3127, 3091, 3029, FNA, FNC or FNE specified | IHA | IHA |
| PCI: ICD9 36.01-36.05  FNG specified 3080 |  | IHA |
| **Cardiopulmonary resuscitation in the**  **community** | |  |
| Numbers | LSH | LSH |
| Uptake | LSH | LSH |
| **Acute myocardial infarction** |  |  |
| Hospital Resuscitation | LSH | LSH |
| Thrombolysis | Assume zero | IHA |
| Aspirin | Assume zero | LSH |
| Beta blockers | Assume zero | LSH |
| ACE inhibitors | Assume zero | LSH |
| Primary CABG surgery | Assume zero | LSH |
| Primary PCI (angioplasty) | Assume zero | LSH |

| **Angina pectoris: unstable** |  |  |
| --- | --- | --- |
| Prevalence | Assume zero | LSH |
| Platelet IIB/IIIA Inhibitors | Assume zero | LSH |
| Aspirin alone | Assume zero | LSH |
| Aspirin & Heparin | Assume zero | LSH |
| Primary CABG surgery | Assume zero | NRMI |
| Primary PCI (angioplasty) | Assume zero | IHA |
| **Secondary prevention following AMI** | |  |
| Aspirin | Assume zero | IHA |
| Beta blockers | Assume zero | IHA |
| ACE inhibitors | Assume zero | LSH |
| Statins | Assume zero | LSH |
| Warfarin | Assume zero | NHANES 1999-2000 |
| Rehabilitation | Assume zero | LSH |
| **Secondary prevention following CABG or PCI** | |  |
| Aspirin | Assume zero | LSH |
| Beta blockers | Assume zero | LSH |
| ACE inhibitors | Assume zero | LSH |
| Statins | Assume zero | LSH |
| Warfarin | Assume zero | LSH |
| Rehabilitation | Assume zero | LSH |
| **Congestive Heart Failure** |  |  |
| ACE inhibitors | Assume zero | LSH |
| Beta blockers | Assume zero | LSH |
| Spironolactone | Assume zero | LSH |
| Aspirin | Assume zero | IHA |
| Statins | Assume zero | IHA |
| **Treatment for chronic angina** |  |  |
| CABG surgery | Assume zero | IHA |
| PCI (angioplasty) | Assume zero | IHA |
| **Community angina pectoris: total** |  |  |
| Prevalence |  | IHA |
| Aspirin | Assume zero | IHA |
| Statins | Assume zero | IHA |
|  |  |  |

| **Community Chronic heart failure** |  |  |
| --- | --- | --- |
| Prevalence |  | IHA |
| ACE inhibitors | Assume zero | LSH  LSH  NHANES 1999-2000 |
| Beta blockers | Assume zero |
| Spironolactone | Assume zero |
| Aspirin | Assume zero | LSH  LSH |
| Statins | Assume zero |
|  |  |  |
| **Hypertension** |  |  |
| Prevalence | IHA | IHA |
| Treated (%) | IHA | IHA |
| **Statins for primary prevention** |  |  |
| Hypercholesterolemia (%) | Assume zero | IHA  LSH |
| Treated (%) | Assume zero |
| **Population risk factor prevalence** | |  |
| Current smoking | IHA | IHA |
| Systolic blood pressure | IHA | IHA |
| Cholesterol | IHA | IHA |
| Physical activity | IHA | IHA |
| Obesity (BMI) | IHA | IHA |
| Diabetes | IHA | IHA |
| *Key*  ACE denotes angiotensin-converting enzyme, AMI acute myocardial infarction, CABG coronary artery bypass graft surgery, ICD International Classification of Diseases, PCI percutaneous coronary intervention, NRMI National Registry of Myocardial Infarction, NHANES National Health and Nutrition Examination Survey, IHA Icelandic Heart Association and LSH National University Hospital in Reykjavík. | | |

# Table 4. Clinical efficacy of interventions: relative risk reductions obtained from meta-analyses, and randomised controlled trials^a^

| **Treatments** | **Relative Risk Reduction**  (95% CI) | **Comments** | **Source paper:**  **First author (year), notes** |
| --- | --- | --- | --- |
| **Acute myocardial infarction** | |  |  |
| Thrombolysis | 31%  (95% CI: 14, 45) | <55 yrs: OR=0.692; RRR=30.8 (95% CI: 14-45)  55-64 yrs: OR=0.736; RRR=26.4 (95% CI: 17-40)  65-74 yrs: OR=0.752; RRR=24.8 (95% CI: 15-37)  >75 yrs: OR=0.844; RRR=15.6 (95% CI: 4-30) | Estess (2002)^19^ [updated FTT] |
| Aspirin | 15%  **(**95% CI: **11, 19)** | **OR=0.85 (**95% CI: **0.81, 0.89). RRR 15% (**95% CI: **11,19) page 75:outcome is vascular and nonvascular deaths** | **Antithrombotic Trialists' Collaboration (2002)^12^** |
| Primary angioplasty STEMI | **32%**  **(**95% CI: **5, 50)** | **OR 0.68 (**95% CI: **0.50, 0.95). RRR 32% (**95% CI: **5,50) outcome compares primary angioplasty to thrombolytics, not specific to STEMI, in results on page 3.** | **Cucherat (2003)**.^20^ |
| Primary PCI non-STEMI | **32%**  **(**95% CI: **5, 51)** | **OR 0.65 (**95% CI: **0.49, 0.95). RRR 32% (**95% CI: **5,51) for cardiovascular death on page 917. [RRR for cardiovascular death or MI was 26 (**95% CI: **3,44) and was 24 (**95% CI: **0,42) for any death]** | **RITA 3 (Fox 2005).^21^** |
| Primary CABG surgery | 20%  **(CI: 16, 24)** | **OR 0.61 (95% CI: 0.48, 0.77). RRR 39% (95% CI: 23,52) on page 565, 0-5 yr mortality. According to later data from MASS-II trial this might be an overestimation. Therefore we estimated the RRR to 20%** | **Yusuf (1994).^22^**  Hueb (2004).**^23^** |
| Beta blockers | 4%  (95% CI: -8, 15) | OR 0.96 (95% CI: 0.85, 1.08), RR 4% (95% CI: -8,15) on page 1732. | Freemantle (1999).^24^ |
| ACE inhibitors | 7%  (95% CI: 2, 11) | OR 0.93, (0.89, 0.98), RR 7% (2,11) for 30 day mortality in MI. | ACE Inhibitor Myocardial Infarction Collaborative Group 1998.^25^ |
| **Cardio-pulmonary resuscitation**  **(CPR)** | |  |  |
| Community CPR | 5%  (95% CI: 4, 15.3) | Nichol study reports overall median survival to discharge at 7.4% in this multi-country/site review, page 520  The Model focuses on 30/7 survival. Discharge survival will therefore provide an over-estimate, which we have explicitly addressed by assuming 5% at 30/7.  Rea looks at odds of bystander dispatcher assisted CPR and bystander CPR without dispatch assistance and compares to No bystander CPR. 7265 out-of-hospital arrests attended. OR 0.59 - 0.69 for these two groups which would give RRRs of 41% and 31%. [Consider as crude equivalent of CPR to no CPR comparison]. 15.3% survival to discharge in King-county, WA; consider as maximum value. Use Nichol (1999)^28^ 5% as USA average.  Graham et al 1999 meta analysis of papers 1973 - 1996 report 6.4% at discharge. Assume better in 2000, thus 6.4% at 30/7 OPALS RCT reports only 5.2%.  Data from Swedish Cardiac Arrest Register, and consistent with data with Nichol^32^ and Rea.^33^ | Nichol (1999).^26^  Rea (2001).^27^  Holmberg (1998).^28^ |
| Hospital CPR | 33%  (95% CI: 10, 36) | AMI accounted for 35% of adult total cases. Adult survival to discharge 36% post VF or VT (majority of post AMI cases, only 10.6% post asystole,  Adult survival to discharge 18% overall, but this reflected ALL Medical arrests in hospital. (Varied from 10-36% depending on type of initial rhythm) (Tables 4 & 5 page 55)  Review of 36,000 adults with cardiac arrests in the 253 US/Canadian Hospitals National Registry of CPR. Nadkarni, JAMA, 2006:295 (1) 50-57)  Older article from Tunstall-Pedoe on page 1350 shows survival at 24 hrs to be 32%, discharge to home at 21%, and 1 year survival to be 15% overall. (16% and 8% in general wards, 31% and 16% in coronary care unit (page 1349), etc.  Corroboration: Model assumes that approximately 2% AMI admissions have primary VF (Olmsted County study). This is consistent with RIKS-HIA, suggesting approximately 2.5%  AMI admissions have primary VF/VT. | Nadkarni (2006).^29^  Tunstall-Pedoe (1992).^30^  RIKS-HIA |
| **Secondary prevention in CHD Patients** | | |  |
| Aspirin | 15%  **(**95% CI: **11, 19)** | OR 0.85 (95% CI: 0.49, 0.95), RR **15% (**95% CI: **11, 19**) **outcome is vascular and nonvascular deaths on page 75. This data seems to be appropriate to this outcome in CHD patients** | **Antithrombotic Trialists' Collaboration (2002).^12^** |
| Beta blockers | 23%  (95% CI: 15, 31) | OR 0.77 (95% CI: 0.85, 0.69), 23% (95% CI: 15,31) on page 1734. Odds of death in long-term trials. | Freemantle (1999).^24^ |
| ACE inhibitors | 20%  (95% CI: 13, 26) | OR 0.80 (95% CI: 0.74, 0.87), 20% (95% CI: 13,26) on page 1577, death up to 4 years [endpoint of study looking at those with heart failure or LV dysfunction.] | Flather (2000).^31^ |
| Statins | 22%  (95% CI: 10, 26) | OR=0.78 (95% CI: 0.74—0.84). RRR=22% (95% CI: 10, 26)  RR=0.77 (95% CI: 0.68—0.87). RRR=23% (95% CI: 13,30) in those with other CHD | Cholesterol Treatment Trialists’ Collaborators (2005).^32^ |
|  |  | OR=0.77 (95% CI: 0.71-0.83). RRR=23% (95% CI: 17, 29)  Wilt (2004) Section CHD mortality, page 1430. | Wilt (2004).^33^ |
| Warfarin | 22%  (95% CI: 13, 31) | OR=0.78 (95% CI: 0.67-0.90), RRR=22% (95% CI: 10, 33)  Meta-analysis looking at oral anticoagulant therapy in coronary artery disease (31 trials about 18,000 patients) by intensity of INR control: High intensity (INR>2.8) warfarin vs. control for outcome of death had OR of 0.78(95% CI: 0.69-0.87) corresponding to a RRR of 22% (95% CI: 13, 31); Moderate intensity warfarin (INR 2-3.0) vs. control had OR of 0.82 (95% CI: 0.23-2.33) not significant but corresponding RRR of 18% (95% CI: -133, 77) | Anand and Yusuf (1999).^34^  Lau (1992).^35^ Table 1, page 253 (anticoagulants). |

| Rehabilitation | 26%  (95% CI: 10, 39) | OR= 0.74 (95% CI: 0.61-0.90), RRR = 26% (95% CI: 10, 39) in Fig 1, page 685 Taylor reference. | Taylor (2004).^36^ |
| --- | --- | --- | --- |
| **Chronic Angina** | |  |  |
| CABG surgery years  0-5 | 39% **(**95% CI: **23, 52)** | OR= 0.61 (95% CI: 0.48-0.77), RR **39% (**95% CI: **23,52) on page 565, 5 yr mortality.** | Yusuf (1994).^22^ |
| CABG surgery years 6-10 | 32%  **(**95% CI: **2, 30)** | OR= 0.83 (95% CI: 0.70-0.98), RR **17 (**95% CI: **2,30) on page 565, 10 yr mortality,** OR= 0.68 (95% CI: 0.56-0.83), RR **32 (**95% CI: **17,44) on page 565, 7 yr mortality CABG compared to medical treatment. This may overestimate benefit, because control groups in 1980s were not on all the modern medical therapies now available.** | Yusuf (1994).^22^ |
| Angioplasty in chronic angina, with stents | 0% | No RRR according to the COURAGE trial and the meta-analysis by Cecil et al. Accordingly we estimated the effectiveness of PCI in patients with stable angina to zero. | COURAGE RCT (2007)^37^: Comparison between PCI vs. optimal medical therapy in patients with stable CAD.  Cecil (2008),^38^: meta-analysis comparing PCI with medical therapy in patients with stable CAD. |
|  |  | Maximum benefit, assume equivalent to CABG surgery for two vessel disease CABG, OR 0.84, (RR 16% 2, 30) 5 year survival 88% in controls.  Minimum assumption: NIL benefit. | Yusuf (1994) ^22^, Pocock (1995),^39^ no difference between PTCA and CABG as initial revasc procedure. Ditto Bucher (2000).^40^ |
| Aspirin | 15%  **(**95% CI: **11, 19)** | OR= 0.85 (95% CI: 0.81-0.89), RR **15% (**95% CI: **11, 19) outcome is vascular and nonvascular deaths on page 75.** | **Antithrombotic Trialists' Collaboration (2002).^12^** |
| Statins | **22%**  **(**95% CI: **10-26)** | **RR=0.78 (**95% CI: **0.74-0.84). RRR=22% (**95% CI: **10, 26)**  **RR=0.77 (**95% CI: **0.68-0.87). RRR=23% (**95% CI: **13,30) in those with other CHD.** | **Cholesterol Treatment Trialists’ Collaborators (2005).^32^** |

| **Unstable Angina** | | |  | |  |
| --- | --- | --- | --- | --- | --- |
| Aspirin alone | | 15%  **(**95% CI: **11, 19)** | OR= 0.85 (95% CI: 0.81-0.89), RR **15% (**95% CI: **11,19) outcome is vascular and nonvascular deaths on page 75. Assume appropriate for unstable angina patients.** | | **Antithrombotic Trialists' Collaboration (2002).^12^** |
| Aspirin & Heparin | | 33%  (95% CI: -2,56) | OR 0.67 (95% CI: 0.48, 1.02) RR 33% (95% CI: -2, 56) in Table 3. The study outcome is composite MI death and non- fatal MI, compares those on ASA + Heparin to ASA only. | | Oler (1996).^41^ |
| Platelet glycoprotein IIB/IIIA inhibitors | | 9%  (95% CI: 2,16) | RR 0.91 (95% CI: 0.84, 0.98) RR 9% (95% CI: 2,16) study looked at acute coronary syndrome without persistent ST elevation. | | Boersma (2002).^42^ |
| Primary PCI Non-STEMI | | 32%  **(**95% CI: **5-51)** | **OR 0.68 (**95% CI: **0.49, 0.95). RRR 32% (**95% CI: **5, 51) for**  **Cardiovascular deaths, table 3.** | | **RITA 3 (Fox 2005).^21^** |
| Primary CABG surgery | | **43%**  **(95%CI: 19,60)** | OR 0.57 (95% CI: 0.40, 0.81). RR 43% (95% CI: 19,60) reduction in mortality at 5 years in those with class III/IV angina, table 4, page 566. **This may overestimate benefit, because control groups in 1980s were not on all the modern medical therapies now available.** | | Yusuf (1994). **^22^** |
| **Heart failure in patients requiring hospitalization** | | | |  |  |
| ACE inhibitors &  angiotensin II receptor blockers (ARBs) | 20% (95% CI: 13,26) | | **OR 0.80 (**95% CI: **0.74, 0.87). RR** 20% (95% CI: 13,26) on page 1577, [death up to 4 years was study endpoint for those with heart failure or LV dysfunction]. | | Flather (2000).^31^  Lakdhar (2008) |
| Beta blockers | 35% (95% CI: 26,43) | | **OR 0.65 (**95% CI: **0.57, 0.74).**  RR 35% (95% CI: 26,43): all cause mortality. | | Shibata (2001).^43^ |
| Spironolactone | 30%  (95% CI: 18, 41) | | **OR 0.70 (**95% CI: **0.59, 0.82).**  RR 30% (95% CI: 18, 41) in those that had at least one cardiac related hospitalization. [31% (95% CI: 18-42) in entire study population of those with CHF, page 711]. [only half patients tolerated this long term]. | | Pitt (1999).^44^ |
| Aspirin | 15%  **(**95% CI: **11,19)** | | OR= 0.85 (95% CI: 0.81, 0.89), RR **15% (**95% CI: **11,19) outcome is vascular and nonvascular deaths on page 75.** | | **Antithrombotic Trialists' Collaboration (2002).^12^** |
| Statin | 0% | | Assume Zero effect. | | GISSI3 RCT Lancet 2008.**^45^** |
| **Heart failure in the community** | | |  | |  |
| ACE inhibitors &  angiotensin II receptor blockers (ARBs) | 20%  (95% CI: 13,26) | | **OR 0.80 (**95% CI: **0.74, 0.87).** RR 20% (95% CI: 13,26) on page 1577, death up to 4 years [in those with heart failure or LV dysfunction]. | | Flather (2000).^31^  Lakhdar (2008) |
| Beta blockers | 35% (95% CI: 26,43) | | **OR 0.65 (**95% CI: **0.57, 0.74).** RR 35 (95% CI: 26,43). Section 3.3 page 353. | | Shibata (2001).^43^ |
| Spironolactone | 31%  (95% CI: 18, 42) | | **OR 0.69 (**95% CI: **0.58, 0.82).** RR 31% (95% CI: 18-42) in entire study population consisting of those with CHF, page 711 [30 (95% CI: 18, 41) in those with a cardiac related hospitalization]. | | Pitt (1999).^44^ |
| Aspirin | 15%  **(**95% CI: **11, 19)** | | OR= 0.85 (0.81, 0.89), RR **15% (11,19) outcome is vascular and nonvascular deaths on page 75. Assume appropriate for patients with CHF due to CHD.** | | **Antithrombotic Trialists' Collaboration (2002).^12^** |
| Statins | **0%** | | Assume Zero effect. | | **GISSI3 RCT Lancet 2008.^45^** |
| **Hypertension treatment** | | |  | |  |
|  | 13%  (95% CI: 6,19) | | **OR 0.87 (**95% CI: **0.81, 0.94).** RRR 13% (95% CI: 6, 19) in those with high blood pressure without disease at entry. [RRR 29% (95% CI: 17, 37) those with average blood pressure and CHD, treated with ACE]. | | Law (2003).^46^ |
| **Therapies for primary prevention of raised cholesterol** | | |  | |  |
| Statins | 35%  (95% CI: 11, 52) | | **OR 0.65 (**95% CI: **0.48, 0.89).** 35% (95% CI: 11,52) for chd mortality (only trials using statins), figure 3 on page 4. | | Pignone (2000).^47^ |
| Gemfibrozil | 7%  (95% CI: -8, 19) | | OR 0.93 (95% CI: 0.81, 1.08); RRR 7% (95% CI: -8, 19). | | Studer (2005).^48^ |
| Niacin | 5%  (95% CI: -10, 18) | | OR 0.95 (95% CI: 0.82, 1.10); RRR 5% (95% CI: -10, 0.18). | | Studer (2005).^48^ |

**^a^**Relative Risk Reduction calculated as 1- Odds Ratio

# Table 5. Data sources for treatment uptake levels in Iceland in 2006: Medical and surgical treatments included in the model

| Treatments | **Treatment Uptake**  **in 2006 *(as reported in source^a^)*** | **Source (year)** |
| --- | --- | --- |
| **Acute myocardial infarction** | |  |
| Thrombolysis | 15.5% | IHA  LSH  LSH  LSH  LSH  LSH |
| Aspirin | 87.6% | LSH |
| Primary angioplasty | 75.3% | LSH |
| Primary CABG | 7.9% | LSH |
| Intravenous and /or oral  Beta blockers | 82.6% | LSH |
| ACE inhibitors | 40.4% | LSH |
| Cardio-pulmonary resuscitation | |  |
| In the Community | 100%***^b^*** | LSH |
| In Hospital | 100%***^b^*** | LSH |
|  | | |
| **Secondary Prevention in CHD Patients** | | |
| Aspirin | 91.4% | IHA |
| Beta blockers | 84.6% | IHA |
| ACE inhibitors | 43.9% | LSH |
| Statins | 94.5% | LSH |
| Warfarin | 9% | NHANES 1999-2000 |
| Rehabilitation | 10% | LSH |
|  |  |  |
| **Chronic Angina** | | |
| CABG surgery | 100%***^c^*** | IHA |
| Angioplasty | 100%***^c^*** | IHA |
| Aspirin in community | 50.8% | IHA |
| Statins in community | 61.5% | IHA |
|  | | |
| **Unstable Angina** | | |
| Aspirin & Heparin | 90% | LSH  LSH  LSH  NRMI  IHA |
| Aspirin alone | 4.8% | LSH |
| Platelet glycoprotein IIB/IIIA inhibitors | 31% | LSH |
| CABG surgery for UAP | 20% | NRMI |
| Angioplasty for UAP | 42% | IHA |
|  | | |
| **Heart Failure including a hospital admission** | | |
| ACE inhibitors | 36% | LSH |
| Beta blockers | 41% | LSH |
| Spironolactone | 19% | LSH |
| Aspirin | 68.7% | IHA |
| Statins | 50.4% | IHA |
| **Heart Failure in the community** | | |
| ACE inhibitors | 30% | LSH |
| Beta blockers | 58% | LSH |
| Spironolactone | 8% | NHANES 1999-2000 |
| Aspirin | 88% | LSH |
| Statins | 28% | LSH |
| **Hypertension treatments** | 65% | IHA |
| **Hyperlipidemia - 1’ prevention** | |  |
| Statins | 4% | IHA |
| Gemfibrozil | 0% | LSH |
| Niacin | 0% | LSH |

^a^Uptake percentages as reported in source papers. Values may differ from those in Table 1 of manuscript, which report weighted averages for ALL age groups 25-74 years included in the Model.

**^b^** 100% taking into account the actual number of CPR patients in the community and in hospital.

**^c^** 100% taking into account the actual number of patients referred for PCI and CABG

LSH = Landspitali National University Hospital

IHA = Icelandic Heart Association

NRMI = National Registry of Myocardial Infarction

NHANES = National Health and Nutrition Examination Survey,

Table 6. Age-specific case fatality rates for each patient group

| **GROUP** | **AMI** | | **Post AMI** | | **Unstable** | | **CABG** | **Angioplasty** | | | **Heart** | **Failure** | | **Hypertension** | | **Hypercholesteraemia** | |
| --- | --- | --- | --- | --- | --- | --- | --- | --- | --- | --- | --- | --- | --- | --- | --- | --- | --- |
|  | |  | | | | **Angina** | **surgery** | |  | ***Hospital*** | | | ***Community*** | |  | |  |
| *Interval* | | **30 day** | | **One year^a^** | | **One year^a^** | **One year^a^** | | **One year^a^** | **One year** | | | **One year** | | **One year** | | **One year** |
| **Mean** | | 0.084 | | 0.051 | | 0.069 | 0.020 | | 0.016 | 0.246 | | | 0.081 | | 0.010 | | 0.006 |
| **MEN** | |  | |  | |  |  | |  |  | | |  | |  | |  |
| **25-34** | | *0.011* | | 0.008 | | 0.016 | 0.003 | | 0.003 | 0.034 | | | 0.011 | | 0.000 | | 0.000 |
| **35-44** | | *0.012* | | 0.009 | | 0.024 | 0.005 | | 0.005 | 0.068 | | | 0.022 | | 0.001 | | 0.001 |
| **45-54** | | *0.023* | | 0.017 | | 0.034 | 0.007 | | 0.007 | 0.096 | | | 0.032 | | 0.002 | | 0.002 |
| **55-64** | | *0.054* | | 0.034 | | 0.056 | 0.012 | | 0.012 | 0.140 | | | 0.045 | | 0.006 | | 0.006 |
| **65-74** | | *0.101* | | *0.073* | | 0.070 | 0.023 | | 0.025 | 0.283 | | | 0.093 | | 0.014 | | 0.014 |
| **75-84** | | *0.164* | | *0.122* | | 0.091 | 0.042 | | 0.042 | 0.337 | | | 0.111 | | 0.035 | | 0.035 |
| **85+** | | *0.279* | | 0.189 | | 0.118 | 0.075 | | 0.074 | 0.418 | | | 0.138 | | 0.094 | | 0.094 |
|  | |  | |  | |  |  | |  |  | | |  | |  | |  |
| **WOMEN** | |  | |  | |  |  | |  |  | | |  | |  | |  |
| **25-34** | | *0.011* | | *0.004* | | *0.016* | *0.003* | | *0.003* | 0.034 | | | 0.011 | | 0.000 | | 0.000 |
| **35-44** | | *0.013* | | *0.006* | | *0.024* | *0.005* | | *0.005* | 0.068 | | | 0.022 | | 0.001 | | 0.001 |
| **45-54** | | *0.026* | | *0.010* | | *0.034* | *0.007* | | *0.007* | 0.096 | | | 0.032 | | 0.001 | | 0.001 |
| **55-64** | | *0.061* | | *0.019* | | *0.056* | *0.012* | | *0.012* | 0.140 | | | 0.045 | | 0.002 | | 0.002 |
| **65-74** | | *0.114* | | *0.084* | | *0.070* | *0.023* | | *0.027* | 0.222 | | | 0.081 | | 0.007 | | 0.007 |
| **75-84** | | *0.167* | | *0.116* | | *0.091* | *0.042* | | *0.039* | 0.289 | | | 0.094 | | 0.021 | | 0.021 |
| **85+** | | *0.267* | | *0.177* | | *0.118* | *0.075* | | *0.061* | 0.368 | | | 0.121 | | 0.079 | | 0.079 |
|  | |  | |  | |  |  | |  |  | | |  | |  | |  |
| ***Source*** | | **Medicare** | | **Medicare** | | **Van Domberg^56^** | **Medicare** | | **Medicare** | **Medicare** | | | **Medicare** | | **NHANES & Vital Statistics** | | |

^a^excluding heart failure patients (already considered within heart failure groups)

**Table 7. Specific Beta Coefficients for Major Risk Factors:** **Data sources, values and comments**

Estimated β coefficients from multiple regression analyses for the relationship between absolute changes in population mean risk factors and % changes in coronary heart disease mortality for men and women, stratified by age.

| **Systolic Blood Pressure** | **Age groups (years)** | | | | |  |
| --- | --- | --- | --- | --- | --- | --- |
|  | 25-44 | 45-54 | 55-64 | 65-74 |  | |
| **Men** (hazard ratio per 20 mmHg) | **0.49** | **0.49** | **0.52** | **0.58** |  | |
| **Men** (log hazard ratio per 1 mmHg) | **-0.036** | **-0.035** | **-0.032** | **-0.027** |  | |
| Min | -0.029 | -0.028 | -0.026 | -0.022 |  | |
| Max | -0.043 | -0.042 | -0.039 | -0.032 |  | |
| **Women** (hazard ratio per 20 mmHg) | **0.40** | **0.40** | **0.49** | **0.52** |  | |
| **Women (log hazard ratio per 1 mmHg)** | **-0.046** | **-0.046** | **-0.035** | **-0.032** |  | |
| Min | -0.037 | -0.037 | -0.028 | -0.026 |  | |
| Max | -0.055 | -0.055 | -0.042 | -0.039 |  | |
| Source: Prospective studies collaborative meta-analysis, Lancet 2002^13^  *UNITS: % mortality change per 20 mmHg change in Systolic BP | | | | | |  |

| **Cholesterol** | **Age groups (years)** | | | | | | | | |  | |
| --- | --- | --- | --- | --- | --- | --- | --- | --- | --- | --- | --- |
|  | 25-44 | 45-54 | | 55-64 | | | 65-74 | |  | | |
| **Mortality reduction per 1 mmol/l**  Men | **0.55** | **0.53** | | **0.36** | | | **0.21** | |  | | |
| Women | **0.57** | **0.52** | | **0.35** | | | **0.23** | |  | | |
| **Log coefficient**  Men | -0.799 | -0.755 | | -0.446 | | | -0.236 | |  | | |
| Women | -0.844 | -0.734 | | -0.431 | | | -0.261 | |  | | |
| Source: Prospective studies collaborative meta-analysis, Lancet 2007.^49^  Body Mass Index (BMI) | **Age groups (years)** | | | | | | | | |  | |
|  | <44 | | 45-59 | | 60-69 | 70-79 | |  | | |  |
| Risk reduction per 1 kg/m^2^:  James Asia Pacific data | 0.1100 | | 0.0900 | | 0.0500 | 0.0400 | |  | | |  |
| Asia Pacific age gradient therefore: | 1.22 | | **1.00** | | 0.56 | 0.44 | |  | | |  |
| Bogers relative risks,  CHD deaths per 5 kg/m^2^ |  | | **1.16** | |  |  | |  | | |  |
| Age specific relative risks per 1 kg/m^2^,  Applying age gradients from James et al | 1.04 | | **1.03** | | 1.02 | 1.01 | |  | | |  |
| **Men & Women, log coefficients*** | **0.0363** | | **0.0297** | | **0.0165** | **0.0132** | |  | | |  |
| Minimum values | 0.0255 | | 0.0209 | | 0.0116 | 0.0093 | |  | | |  |
| Maximum values *(from James et al)* | 0.0466 | | 0.0381 | | 0.0212 | 0.0169 | |  | | |  |
| Source: Bogers et al. 2006,^50^ James et al. 2004.^51^ *UNITS: % mortality change per 1 kg/m^2^ change in BMI  Strengths: Large number of studies included. Adjusted for blood pressure, total cholesterol, and physical activity. 95% CIs also provided. Limitations: Observational data; age gradient applied from James study. | | | | | | | | | |  | |

**Table 8. Relative Risks Used in the Icelandic IMPACT Model for Smoking, Diabetes and Physical Inactivity for Coronary Heart Disease Mortality.** (Best, Minimum and Maximum Estimates from the InterHeart Study**^a^**)

|  | **Both sexes** | | **Men** | | **Women** | |
| --- | --- | --- | --- | --- | --- | --- |
|  | **Young** | **Old** |  | **>55 years** |  | **> 65 years** |
| Lifestyle factors |  |  |  |  |  |  |
| **Smoking** | **3.33 (2.86-3.87)** | **2.44 (2.10-2.84)** | **3.33 (2.80-3.95)** | **2.52 (2.15-2.96)** | **4.49 (3.11-6.47)** | **2.14 (1.35-3.39)** |
| **Fruit and vegetables** | 0.69 (0.58-0.81) | 0.72 (0.6-0.85) | 0.72 (0.59-0.88) | 0.77 (0.64-0.93) | 0.62 (0.44-0.87) | 0.55 (0.38-0.80) |
| **Exercise** | **0.95 (0.79-1.14)** | **0.79 (0.66-0.94)** | **1.02 (0.83-1.25)^b^** | **0.79 (0.66-0.96)** | **0.74 (0.49-1.10)** | **0.75 (0.46-1.22)** |
| **Alcohol** | 1.00 (0.85-1.17) | 0.85 (0.73-1.00) | 1.03 (0.87-1.23) | 0.86 (0.73-1.01) | 0.74 (0.41-1.31) | 0.83 (0.49-1.42) |
| **Hypertension** | 2.24 (1.93-2.60) | 1.72 (1.52-1.95) | 1.99 (1.66-2.39) | 1.72 (1.49-1.98) | 2.94 (2.25-3.85) | 1.82 (1.39-2.38) |
| **Diabetes** | **2.96 (2.40-3.64)** | **2.05 (1.71-2.45)** | **2.66 (2.04-3.46)** | **1.93 (1.58-2.37)** | **3.53 (2.49-5.01)** | **2.59 (1.78-3.78)** |
| **Abdominal obesity** | 1.79 (1.52-2.09) | 1.50 (1.29-1.74) | 1.83 (1.52-2.20) | 1.54 (1.30-1.83) | 1.58 (1.14-2.20) | 1.22 (0.88-1.70) |
| **Psychosocial** | 2.87 (2.19-3.77) | 2.43 (1.86-3.18) | 2.62 (1.91-3.60) | 2.45 (1.82-3.29) | 3.92 (2.26-6.79) | 2.31 (1.22-4.39) |
| **High ApoB/ApoA1 ratio** | 4.35 (3.49-5.42) | 2.50* (2.05-3.05) | 4.16 (3.19-5.42) | 2.51 (2.00-3.15) | 4.83 (3.19-7.32) | 2.48 (1.60-3.83) |

(and see Introduction for a worked example)

**Yusuf, InterHeart Study. Lancet 2004.^14^ *Odds ratios for relative effect of risk factors*** *(99% Confidence Intervals, NOT 95%)*

Smoking, adverse lipid profile, hypertension, and diabetes had a greater relative effect on risk of acute myocardial infarction in younger than older individuals

**^a^**Global InterHeart values were used in the Icelandic IMPACT Model

^b^The InterHeart study quoted a value of only 1.02 for exercise in men aged <55 years. This was clearly an outlier. We have therefore assumed a value of 0.77 in line with men and women in the other age groups, and consistent with most other studies.

gg g

# Table 9. Iceland Impact Model Risk Factor Methodology: Rationale for choice of regression or PARF approaches for specific risk factors

Modelling treatment effects appears reasonably precise, because each treatment has a meta-analysis with a fairly well quantified efficacy value, plus 95% confidence intervals.

Quantifying the mortality reduction attributable to the change in a specific risk factor remains a less precise science. This table explains the rationale for choosing the best approach for each risk factor: regression based on absolute change in the risk factor*, regression based on relative change in the risk factor*, or population attributable risk fraction (PARF).

We also specify the best data source for each.

*Absolute and Relative beta regression approaches are illustrated earlier in the Supplementary Appendix.

An absolute beta regression coefficient quantifies the CHD mortality reduction for each UNIT change in risk factor, e.g. mmHg change for BP, or mg/dl change for cholesterol

A RELATIVE beta regression coefficient quantifies the CHD mortality reduction for each % relative change in risk factor, e.g. a 12 mmHg fall in SBP, from 120 mmHg to 108 mmHg, would represent a **10%** relative decrease (12/120).

| **Risk Factor** | **Source** | Strengths | **Limitations** | **Comments and recommend-**  **ation** | **DPP value in Icelandic Model** (contribution to total CHD mortality fall) |
| --- | --- | --- | --- | --- | --- |
| **Blood pressure** |  |  |  |  |  |
| 1. Systolic BP: regression using absolute beta approach | PSC 2002^13^ | Large meta-analyses include Swedish and European data. Age and sex stratified.  SBP preferable to  DBP, because stronger relationship with CHD deaths. | Observational data- assume complete reversibly of risk. | CURRENT APPROACH  Supersedes relative approach. | 72 (24.3%) |
| 2. PARF | Midspan | Original approach in Scottish IMPACT Model. | Sensitive to reference value and category cut-offs. Estimated DPPs always appeared very low. | Obsolete | - |

| **Cholesterol** |  |  |  |  |  |
| --- | --- | --- | --- | --- | --- |
| 1. Regression using absolute Beta | Law et al, meta-analysis | Large meta-analysis, split by age and sex; cohort and RCT results very consistent; supported by more recent reviews | Published in 1994 | CURRENT APPROACH | 95 (32.3%) |
| 2. PARF using quintiles | Midspan | Used in 1996 | Sensitive to reference value and category cut-offs. | Obsolete since 1997 | - |
| **BMI** |  |  |  |  |  |
| 1.Regression using absolute Beta | Bogers et al 2006^.50^ | Large meta-analysis with US data, Broadly consistent with Asian and PSC analyses; age-splits taken from James et al. Adjusted for major confounders: smoking, cholesterol, blood pressure, and physical activity | An “upstream” CHD risk factor. CHD risk partly or wholly mediated through “downstream factors: BP, cholesterol and impaired glucose tolerance. DPP values consistent with earlier US studies. | CURRENT APPROACH  Potential confounding addressed by using this adjusted value | -13 (-4.4%) |
| 2. PARF using OBESITY quintiles | Inter-  Heart^52^ | Large, global study including data from Sweden | Sensitive to reference value and category cut-offs. Under-estimation likely. | An arbitrary approach to a continuous variable.  Superseded | -22  (-7.4%) |

| **Smoking** |  |  |  |  |  |
| --- | --- | --- | --- | --- | --- |
| 1. PARF | Inter-  Heart^14^ | Log linear.  InterHeart large, global study including Swedish data.  RRs consistent with other studies. Appropriate for a dichotomous variable. | Regression approach might provide useful alternative approach? | CURRENT APPROACH | 65 (22.0%) |
| 2. Regression using absolute beta | Vartiainen 1994^53^ | Used in earlier IMPACT Models. Result consistent with PARF approach. | Not dichotomous. Not log-linear | Superseded | 148 (50.0%) |

| **Diabetes** |  |  |  |  |  | |  | | |
| --- | --- | --- | --- | --- | --- | --- | --- | --- | --- |
| 1. PARF approach | Inter  Heart^14^ | Large, global study including Swedish data.  RRs consistent with other studies. Appropriate method for dichotomous variable. | Case control study, albeit huge. | CURRENT APPROACH | -14 (-4.6%) | |  | | |
| 2. Regression approach | - | - | Appropriate Betas not identified, and methodologically dubious | Not attempted | - | |  | | |
|  |  |  |  |  |  |  | |  |  |
| **Physical activity** |  |  |  |  |  | |  | | |
| 1. PARF approach | Inter-  Heart^14^ | Large, global study including US data.  RRs consistent with other studies. Appropriate method for dichotomous variable. | Alternative PARF methods possible. Important to use independent RR values. (Aim to examine activity sub-categories in future studies) | CURRENT APPROACH | 16 (5.4%) | |  | | |
| 2. Regression approach | - | - | Appropriate Betas do not exist, and methodologically dubious | Not attempted | - | |  | | |
|  |  |  |  |  |  | |  | | |

| Table 10. Main Assumptions and Overlap Adjustments Used in the Icelandic IMPACT Model | | |
| --- | --- | --- |
| **Treatment category** | Assumptions and overlaP Adjustments |  |
| Efficacy of PCI in angina | Assumed equivalent to CABG surgery for two vessel disease (maximum estimate), or equal to medical therapy (minimum estimate) | Sculpher (1994).^54^  Folland (1997).^55^  Yusuf (1994).^22^ |
| Angina in the community | Start with the total patient numbers with angina in the community, based on INTERGENE prevalence^a^  Then deduct patients counted elsewhere:  -Patients already treated for unstable angina in hospital,  -50% of those receiving CABG for angina  -50% of those receiving secondary prevention post AMI/post CABG/Post Angioplasty, | Capewell (2000).^1^ |
| Hypertension treatment: overlaps with other CHD patient groups | Total hypertensive patient numbers in community calculated, then deduct:  -50% of post AMI patients  -50% of community angina patients  -50% of community heart failure patients | NHANES 1999- 2000 |
| Fall in population blood pressure | Estimate the number of DPPs by hypertension treatment  -Then subtract this from the total DPPs attributed to the secular fall in population BP | Capewell (1999).^2^  Capewell (2000).^1^ |
| AMI denotes acute myocardial infarction, CABG coronary artery bypass graft surgery, CHD coronary heart disease, DPPs deaths prevented or postponed and NHANES National Health and Nutrition Examination Survey.  ^a^Prevalence of angina according to Rose’ questionnaire was established. Validation of the cases reduced the prevalence by half | | |

**REFERENCES**

1. Capewell S, Beaglehole R, Seddon M, McMurray J. Explanation for the decline in coronary heart

disease mortality rates in Auckland, New Zealand, between 1982 and 1993. *Circulation*.

2000;**102**(13):1511-1516.

1. Capewell S, Morrison CE, McMurray JJ. Contribution of modern cardiovascular treatment and risk

factor changes to the decline in coronary heart disease mortality in Scotland between 1975 and 1994.

*Heart*. 1999;**81**(4):380-386.

1. Laatikainen T, Critchley J, Vartiainen E, Salomaa V, Ketonen M, Capewell S. Explaining the decline

in coronary heart disease mortality in Finland between 1982 and 1997. *Am J Epidemiol*.

2005;**162**(8):764-773.

1. Unal B, Critchley JA, Capewell S. Explaining the decline in coronary heart disease mortality in

England and Wales between 1981 and 2000. *Circulation*. 2004;**109**(9):1101-1107.

1. Unal B, Critchley JA, Capewell S. Modelling the decline in coronary heart disease deaths in England

and Wales, 1981-2000: comparing contributions from primary prevention and secondary prevention.

*BMJ*. 2005;**331**(7517):614.

1. Critchley J, Liu J, Zhao D, Wei W, Capewell S. Explaining the increase in coronary heart disease

mortality in Beijing between 1984 and 1999. *Circulation*. 2004;**110**(10):1236-1244.

1. Ford ES, Ajani UA, Croft JB, Critchley JA, Labarthe DR, Kottke TE, Giles WH, Capewell S.

Explaining the decrease in U.S. deaths from coronary disease, 1980-2000. *N Engl J Med*.

2007;**356**(23):2388-2398.

1. Björck L, Rosengren A, Bennett K, Lappas G, Capewell S. Modeling the decreasing coronary heart

disease mortality in Sweden between 1986 and 2002. European Heart Journal. 2009;0:ehn554v1-11.

1. Unal B, Critchley J, Capewell S. IMPACT, a validated, comprehensive coronary heart disease

model. Liverpool, United kingdom: University of Liverpool 2006. (Accessed April 27, at

http://www.liv.ac.uk/PublicHealth/sc/bua/impact.html).

1. Nichol MB, Venturini F, Sung JC. A critical evaluation of the methodology of the literature on

medication compliance. *Ann Pharmacother*. 1999;**33**(5):531-540.

1. Butler J, Arbogast PG, BeLue R, Daugherty J, Jain MK, Ray WA, Griffin MR. Outpatient adherence

to beta-blocker therapy after acute myocardial infarction. *J Am Coll Cardiol*. 2002;**40**(9):1589-1595.

1. Antithrombotic TC. Collaborative meta-analysis of randomised trials of antiplatelet therapy for

prevention of death, myocardial infarction, and stroke in high risk patients. *BMJ*.

2002;**324**(7329):71-86.

1. Lewington S, Clarke R, Qizilbash N, Peto R, Collins R. Age-specific relevance of usual blood

pressure to vascular mortality: a meta-analysis of individual data for one million adults in 61 prospective studies. *Lancet*. 2002;**360**(9349):1903-1913.

1. Yusuf S, Hawken S, Ounpuu S, Dans T, Avezum A, Lanas F, McQueen M, Budaj A, Pais P, Varigos

J, Lisheng L. Effect of potentially modifiable risk factors associated with myocardial infarction in 52 countries (the INTERHEART study): case-control study. *Lancet*. 2004;**364**(9438):937-952.

1. Mant J, Hicks N. Detecting differences in quality of care: the sensitivity of measures of process and

outcome in treating acute myocardial infarction. *BMJ*. 1995;**311**(7008):793-796.

1. Yusuf S. Two decades of progress in preventing vascular disease. *Lancet*. 2002;**360**(9326):2-3.
2. Wald NJ, Law MR. A strategy to reduce cardiovascular disease by more than 80%. *BMJ*.

2003;**326**(7404):1419.

1. Briggs A, Sculpher M, Buxton M. Uncertainty in the economic evaluation of health care technologies: the role of sensitivity analysis. *Health Econ*. 1994;**3**(2):95-104.
2. Estess JM, Topol EJ. Fibrinolytic treatment for elderly patients with acute myocardial infarction. *Heart*. 2002;**87**(4):308-311.
3. Cucherat M, Bonnefoy E, Tremeau G. Primary angioplasty versus intravenous thrombolysis for

acute myocardial infarction. *Cochrane Database Syst Rev*. 2000(**2**):CD001560.

1. Fox KA, Poole-Wilson P, Clayton TC, Henderson RA, Shaw TR, Wheatley DJ, Knight R, Pocock

SJ. 5-year outcome of an interventional strategy in non-ST-elevation acute coronary syndrome: the

British Heart Foundation RITA 3 randomised trial. *Lancet*. 2005;**366**(9489):914-920.

1. Yusuf S, Zucker D,Passamani E, Peduzzi P, Takaro T, Fisher LD, , Kennedy JW, Davis K, Killip T,

Norris R, Morris C, Mathur V, Varnauskas E, Chalmers TC. Effect of coronary artery bypass graft

surgery on survival: overview of 10-year results from randomised trials by the Coronary Artery

Bypass Graft Surgery Trialists Collaboration. *Lancet*. 1994;**344**(8922):563-570.

1. Hueb W, Soares PR, Gersh BJ, César LAM, Luz PL, Puig LB, Martinez EM, Oliveira SA, Ramires

JAF. The medicine, angioplasty, or surgery study (MASS-II): a randomized, controlled clinical trial

of three therapeutic strategies for multivessel coronary artery disease: One-year results. *Journal of*

*the American College of Cardiology*. 2004;**43**(10):1743-1751.

1. Freemantle N, Cleland J, Young P, Mason J, Harrison J. beta Blockade after myocardial infarction:

systematic review and meta regression analysis. *BMJ*. 1999;**318**(7200):1730-1737.

1. ACE Inhibitor Myocardial Infarction Collaborative Group. Indications for ACE inhibitors in the

early treatment of acute myocardial infarction: systematic overview of individual data from 100,000

patients in randomized trials. ACE Inhibitor Myocardial Infarction Collaborative Group. *Circulation*.

1998;**97**(22):2202-2212.

1. Nichol G, Stiell IG, Hebert P, Wells GA, Vandemheen K, Laupacis A. What is the quality of life for

survivors of cardiac arrest? A prospective study. *Acad Emerg Med*. 1999;**6**(2):95-102.

1. Rea TD, Eisenberg MS, Culley LL, Becker L. Dispatcher-assisted cardiopulmonary resuscitation and

survival in cardiac arrest. *Circulation*. 2001;**104**(21):2513-2516.

1. Holmberg M, Holmberg S, Herlitz J, Gardelov B. Survival after cardiac arrest outside hospital in

Sweden. Swedish Cardiac Arrest Registry. *Resuscitation*. 1998;**36**(1):29-36.

1. Nadkarni VM, Larkin GL, Peberdy MA, Carey SM, Kaye W, Mancini ME, Nichol G, Lane-Truitt T,

Potts J, Ornato JP, Berg RA. First documented rhythm and clinical outcome from in-hospital cardiac

arrest among children and adults. *JAMA*. 2006;**295**(1):50-57.

1. Tunstall-Pedoe H, Bailey L, Chamberlain DA, Marsden AK, Ward ME, Zideman DA. Survey of

3765 cardiopulmonary resuscitations in British hospitals (the BRESUS Study): methods and overall

results. *BMJ*. 1992;**304**(6838):1347-1351.

1. Flather MD, Yusuf S, Kober L, Pfeffer M, Hall A, Murray G, Torp-Pedersen C, Ball S, Pogue J,

Moye L, Braunwald E. Long-term ACE-inhibitor therapy in patients with heart failure or leftventricular

dysfunction: a systematic overview of data from individual patients. ACE-Inhibitor

Myocardial Infarction Collaborative Group. *Lancet*. 2000;**355**(9215):1575-1581.

1. Baigent C, Keech A, Kearney PM, Blackwell L, Buck G, Pollicino C, Kirby A, Sourjina T, Peto R,

Collins R, Simes R. Efficacy and safety of cholesterol-lowering treatment: prospective meta-analysis

of data from 90,056 participants in 14 randomised trials of statins. *Lancet*. 2005;**366**(9493):1267-

1278.

1. Wilt TJ, Bloomfield HE, MacDonald R, Nelson D, Rutks I, Ho M, Larsen G, McCall A, Pineros S,

Sales A. Effectiveness of statin therapy in adults with coronary heart disease. *Arch Intern Med*.

2004;**164**(13):1427-1436.

1. Anand SS, Yusuf S. Oral anticoagulant therapy in patients with coronary artery disease: a metaanalysis.

*JAMA*. 1999;**282**(21):2058-2067.

1. Lau J, Antman EM, Jimenez-Silva J, Kupelnick B, Mosteller F, Chalmers TC. Cumulative metaanalysis

of therapeutic trials for myocardial infarction. *N Engl J Med*. 1992;**327**(4):248-254.

1. Taylor RS, Brown A, Ebrahim S, Jolliffe J, Noorani H, Rees K, Skidmore B, Stone JA, Thompson

DR, Oldridge N. Exercise-based rehabilitation for patients with coronary heart disease: systematic

review and meta-analysis of randomized controlled trials. *Am J Med*. 2004;**116**(10):682-692.

1. Boden WE, O'Rourke RA, Teo KK, Hartigan PM, Maron DJ, Kostuk WJ, Knudtson M, Dada M,

Casperson P, Harris CL, Chaitman BR, Shaw L, Gosselin G, Nawaz S, Title LM, Gau G, Blaustein

AS, Booth DC, Bates ER, Spertus JA, Berman DS, Mancini GB, Weintraub WS. Optimal medical

therapy with or without PCI for stable coronary disease. *N Engl J Med*. 2007;**356**(15):1503-1516.

1. Cecil WT, Kasteridis P, Barnes JW, Jr., Mathis RS, Patric K, Martin S. A meta-analysis update:

percutaneous coronary interventions. *The American journal of managed care*. 2008;**14**(8):521-528.

1. Pocock SJ, Henderson RA, Rickards AF, Hampton JR, King SB, 3rd, Hamm CW, Puel J, Hueb W,

Goy JJ, Rodriguez A. Meta-analysis of randomised trials comparing coronary angioplasty with

bypass surgery. *Lancet*. 1995;**346**(8984):1184-1189.

1. Bucher HC, Hengstler P, Schindler C, Guyatt GH. Percutaneous transluminal coronary angioplasty

versus medical treatment for non-acute coronary heart disease: meta-analysis of randomised

controlled trials. *BMJ*. 2000;**321**(7253):73-77.

1. Oler A, Whooley MA, Oler J, Grady D. Adding heparin to aspirin reduces the incidence of

myocardial infarction and death in patients with unstable angina. A meta-analysis. JAMA.

1996;**276**(10):811-815.

1. Boersma E, Harrington RA, Moliterno DJ, White H, Theroux P, Van de Werf F, de Torbal A,

Armstrong PW, Wallentin LC, Wilcox RG, Simes J, Califf RM, Topol EJ, Simoons ML. Platelet

glycoprotein IIb/IIIa inhibitors in acute coronary syndromes: a meta-analysis of all major randomised

clinical trials. *Lancet*. 2002;**359**(9302):189-198.

1. Shibata MC, Flather MD, Wang D. Systematic review of the impact of beta blockers on mortality

and hospital admissions in heart failure. *Eur J Heart Fail*. 2001;**3**(3):351-357.

1. Pitt B, Zannad F, Remme WJ, Cody R, Castaigne A, Perez A, Palensky J, Wittes J. The effect of

spironolactone on morbidity and mortality in patients with severe heart failure. Randomized

Aldactone Evaluation Study Investigators. *N Engl J Med*. 1999;**341**(10):709-717.

1. GISSI-HF investigators. Effect of rosuvastatin in patients with chronic heart failure (the GISSI-HF trial): a randomised, double-blind, placebo-controlled trial. *Lancet*. 2008;**372**(9645):1231-1239.
2. Law M, Wald N, Morris J. Lowering blood pressure to prevent myocardial infarction and stroke: a

new preventive strategy. *Health Technol Assess.* 2003;**7**(31):1-94.

1. Pignone M, Phillips C, Mulrow C. Use of lipid lowering drugs for primary prevention of coronary

heart disease: meta-analysis of randomised trials. *BMJ*. 2000;**32**1(7267):983-986.

1. Studer M, Briel M, Leimenstoll B, Glass TR, Bucher HC. Effect of different antilipidemic agents and

diets on mortality: a systematic review. *Arch Intern Med*. 2005;**165**(7):725-730.

1. Lewington S, Whitelock G, Clarke R, Sherliker P, Emberson J, Halsey J, Qizilbash N, Peto R, Collins R. Blood cholesterol and vascular mortality by age, sex, and blood pressure: a meta-analysis of

individual data from 61 prospective studies with 55 000 vascular deaths. *Lancet*. 2007;**370**(9602):1829-1839.

1. Bogers RP HR, Boshuizen H. et al. Overweight and obesity increase the risk of coronary heart

disease: a pooled analysis of 30 prospective studies. *European Journal of Epidemiology*.

2006;**21**((supplement):107.).

1. James WPT J-LR, Mhurchu CN et al.Overweight and obesity (high body mass index). Comparative

quantification of risk. Global and regional burden of disease attributable to selected major risk

factors.: World Health Organization; 2004.

1. Yusuf S, Hawken S, Ounpuu S, Bautista L,Franzosi MG, Commerford P, Lang CC, Rumboldt Z,

Onen CL, Lisheng L, Tanomsup S, Wangai P, Razak F, Sharma AM, Anand SS.Obesity and the risk

of myocardial infarction in 27,000 participants from 52 countries: a case-control study. *Lancet*

2005;**366**:1640-9.

1. Vartiainen E, Puska P, Pekkanen J, Tuomilehto J, Jousilahti P. Changes in risk factors explain

changes in mortality from ischaemic heart disease in Finland. *BMJ*. **1994**;309(6946):23-27.

1. Sculpher MJ, Seed P, Henderson RA, Buxton MJ, Pocock SJ, Parker J, Joy MD, Sowton E, Hampton

JR. Health service costs of coronary angioplasty and coronary artery bypass surgery: the Randomised

Intervention Treatment of Angina (RITA) trial. *Lancet*. 1994;**344**(8927):927-930.

1. Folland ED, Hartigan PM, Parisi AF. Percutaneous transluminal coronary angioplasty versus medical therapy for stable angina pectoris: outcomes for patients with double-vessel versus single-vessel

coronary artery disease in a Veterans Affairs Cooperative randomized trial. Veterans Affairs ACME Investigators. *J Am Coll Cardiol*. 1997;**29**(7):1505-1511.

1. van Domburg RT, Miltenburg-van-Zijl AJ, Veerhoek RJ, Simoons ML. Unstable angina: good long-term outcome after a complicated early course. J Am Coll Cardiol 1998;31:1534-9.
